# Account of an Organic Injury of the Intestines, Which Produced the Symptoms of Pulmonary Consumption, with the Appearances of the Body on Dissection

**Published:** 1809-08

**Authors:** M. Nacquart


### *Account of an Organic Injury of the Intestines, which produced the Symptoms of Pulmonary Consumption, with the Appearances of the Body on Dissection.* By M. Nacquart.*

M. B. had been early given to the study of the dead languages, and prosecuted them with so much ardor that he neglected the calls of nature. For some years he had accustomed himself to refrain from going to stool at the usual times, and sometimes felt no desire of that description for several weeks.

He becamce subject to very acute cholics, accompanied with obstinate constipation, to which succeeded an habitual diarrhœa.

While the bowels were thus the seat of a slow but unremitting irritation, the chest seemed to be affected, and a sharp cough succeeded, accompanied by an expectoration, at first of mucus, and soon afterwards of pus.

The patient's constitution became daily worse, his weakness increased, the cough was distressing, and the expectoration considerable: the diarrhœa still continued.

Such was the predominance of the symptoms of the disease of the lungs over those of the intestines, that several eminent practitioners of Paris did not hesitate to ascribe the whole symptoms to phthisis, attended with irritation of the bowels.

The treatment of the case, viewed in this light, was attended with no success, and the patient became worse until the 5th of January 1807, when he died at the age of 27, having retained his senses to the last.

*Dissection of the Body.* The chest when struck against resounded throughout its whole extent, with the exception of the middle part and the lower part of the right side. The right lobe had formed some very close adhesions to the pleura; both lobes were sound and firm: mucus, similar to the matter expectorated filled the tracheal artery and the bronchiæ.

The belly was not tumefied: on opening it a very fœtid smell was perceived. The epiploon, loaded with fat, had descended into the pelvis: its back part adhered to the small intestines, and was fringed with a coating of grumous pus, which was accumulated in the circumvolutions of the intestines;

* Journal de Medicine, tom. 31, p. 331.

intestines; the peritoneum contained a great quantity of this purulent liquor; the surface of the small intestines was inflamed.

The colon, bent and contracted, seemed to be smaller in bulk than the small intestines; its passage was in some places almost effaced.

The cæcum was sunk in a cellular texture, hard, blackish, and as it were fleshy: it presented nothing else than a very irregular pouch, with hard and shrivelled edges.

These two intestines having been opened throughout their whole extent, there issued a great quantity of black, purulent, and fœtid sanies, which coloured their internal coat, and this last was thick and like suet.

When carefully examined, the internal surface of the colon and cæcum seemed pierced with holes, which led to very large follicles, filled with black sanies: the transverse section displayed a multitude of black streaks, confined to the surface, and resembling an injection made with ink.

An attentive dissection shewed that this disease was confined to the mucous membrane of the large intestines, the muscular and peritoneal coats having preserved their healthful state.

The liver was sound: its large volume raised the diaphragm considerably, and diminished the right cavity of the chest. The brain was not examined.

I take leave to make the following observations:

1st. Here we have an organic affection of the intestines, without any trace of affection of the lungs. What could be the cause of this change of phænomena? To what can we attribute the striking symptoms of pulmonary consumption which accompanied this disease of the intestines? Perhaps we shall find the cause of it in the nervous communications between these organs, and in the pressure of the lungs by the liver: but our present knowledge of the nature of the mucous membranes, their identity of action, and their frequent simultaneousness of affections, convince me that we ought to ascribe to these membranes the symptoms which have rendered the diagnostic of this disease so uncertain.

2. Here we have to remark two diseases different in their seat and their origin. On the one hand, the disorganization of the mucous membrane of the large intestines, which occurred so far back as the first attacks of disease fifteen years before; on the other hand, the diseased state of the small intestines, which I regard as a consequence of the other.

3. The

3. The isolated state of the cellular textures, in a disease so deep seated, and of such long standing, shews us how important it is not to confound them, Nature having communicated to them functions, structure, and diseases quite different.

4. Finally. The dissection of the body shews that even if this disease had been well known, both in its nature and its seats, it would still have baffled the resources of art.

I am far from offering this observation as unique in its kind; on the contrary, I could adduce several analogous cases from authors who have written on pathology. But although this disease of the intestines may have been already noticed, and although these sympathetic affections of distant organs were long known, I thought them worthy of being once more brought before the public.

